# Exercise Intervention Improves Clinical Outcomes, but the “Time of Session” is Crucial for Better Quality of Life in Breast Cancer Survivors: A Systematic Review and Meta-Analysis

**DOI:** 10.3390/cancers11050706

**Published:** 2019-05-22

**Authors:** Feng Hong, Weibing Ye, Chia-Hua Kuo, Yong Zhang, Yongdong Qian, Mallikarjuna Korivi

**Affiliations:** 1 Exercise and Metabolism Research Center, College of Physical Education and Health Sciences, Zhejiang Normal University, Jinhua 321004, Zhejiang, China; fenghong0313@outlook.com (F.H.); ywbls@zjnu.cn (W.Y.); zhangyong@zjnu.cn (Y.Z.); 2 Department of Sports Sciences, University of Taipei, Taipei 11153, Taiwan; kuochiahua@gmail.com

**Keywords:** physical activity, breast cancer survivors, physical function, social well-being, exercise characteristics

## Abstract

This study examined the effects of exercise intervention on the quality of life (QoL), social functioning (SF), and physical functioning (PF) of breast cancer survivors, and identified the responsible and optimal exercise characteristics for amelioration of outcomes. Randomized controlled trials (RCTs) that adopted exercise intervention and measured the QoL, SF, and PF of breast cancer patients were included. We used meta-analysis to calculate the pooled effect, and meta-regression to identify the responsible exercise characteristics (type, frequency, duration, and time). Subgroup analysis assessed the optimal “time of session” for an improved QoL. The Cochrane risk-of-bias tool was used to determine the quality of studies. In the systematic review, we included 26 RCTs with a total of 1892 breast cancer patients, whilst 18 trials were considered for meta-analysis (exercise = 602; control = 603). The pooled effect showed that exercise intervention substantially improved the QoL (standardized mean difference (SMD) = 0.35; I^2^ = 61%; 95% confidence internal (CI): 0.15–0.54; *p* = 0.0004), SF (SMD = 0.20; I^2^ = 16%; 95% CI:0.08–0.32; *p* = 0.001), and PF (SMD = 0.32; I^2^ = 32%; 95% CI:0.20–0.44; *p* < 0.00001). Meta-regression analysis showed that improved QoL was associated (*p* = 0.041) with the “time of session”. More specifically, sessions conducted for medium-time (>45 to ≤60 min; *p* = 0.03) and longer-time (>60 to 90 min; *p* = 0.005) considerably improved the QoL, whilst shorter-time (≤45 min; *p* = 0.15) did not. To summarize, exercise interventions improved the QoL, SF, and PF of breast cancer survivors, where the “time of session” appeared to be crucial for an effective improvement in the QoL.

## 1. Introduction

Breast cancer is the most commonly diagnosed cancer in women worldwide. The number of newly diagnosed breast cancer patients reached 2.1 million in 2018, accounting for one quarter of female cancer cases [1]. Globally, the number of newly diagnosed breast cancer patients is predicted to reach 3.2 million by 2050 [2,3]. Currently, the highest incidence of breast cancer is found in the developed regions, including Western Europe, Northern Europe, Australia and New Zealand, and North America [1]. Owing to advancements in medical care and treatment, the number of post-treatment cancer survivors has also increased worldwide. The 5-year survival rate for breast cancer patients is 90.2% in the USA, 89.5% in Australia, and 83.2% in China, while it is only 66.2% and 65% for patients in India and Malaysia, respectively [4]. Despite this, after treatment or prognosis, most cancer survivors suffer from a series of psychological and physical adverse symptoms, including nausea, insomnia, depression, anxiety, and fatigue. These side effects eventually impair the social functions (SF) and physical functions (PF) of the women, and lead to a decrease in their overall quality of life (QoL). For instance, negative outcomes, such as decreased expectations for the future, breakdown in social relations, limitation of daily activities, and a decline in self-care ability, were commonly observed in breast cancer survivors [5,6].

In recent decades, there has been a significant increase in the prescription of exercise to cancer survivors as a means to overcome treatment-induced adverse effects [7,8]. Based on the evidence from several randomized controlled trials (RCTs), the American College of Sports Medicine (ACSM) roundtable concluded that exercise training is safe both during and after treatment, and it can improve the QoL and PF, as well as reduce depression, anxiety, and fatigue in breast cancer survivors [9]. However, some studies using exercise intervention reported equivocal results in various clinical outcomes in breast cancer patients. For instance, an RCT of 500 breast cancer survivors reported no change in the QoL (all domains), depression, or fatigue after a 12 month exercise (60 min) program [10]. In contrast, another RCT showed that a 12 week supervised exercise program (>60 min) improved the functional and global health scores linked to the QoL, whilst home-based exercise (30 min) improved only the global health score of the QoL in breast cancer patients [11]. Moreover, resistance exercise (45 min, under an 8-week program) or aerobic exercise (60 min, under a 17-week program) interventions were found to be ineffective in improving the QoL in breast cancer patients [12,13]. To address the type and dose response of exercise (~16 weeks) on PF, Courneya et al. reported that neither high-intensity aerobic exercise (50 to 60 min) nor combined aerobic–resistance exercise (50 to 60 min) were superior to standard aerobic exercise (25 to 30 min) in breast cancer survivors [14].

From these RCTs, it appears that the beneficial effects of exercise on the health outcomes (QoL, SF, PF) should be moderated using one or more exercise characteristics, such as frequency (F), intensity (I), type (T), and time (T) (i.e., the FITT factors). Therefore, to enhance the beneficial effects of exercise, it is necessary to better understand the influences of exercise variables on the QoL, SF, and PF of breast cancer patients. In this context, a systematic review and meta-analysis of 16 RCTs found that shorter workouts improved both the physical and social functioning, whilst longer workouts only improved the PF in patients with cancer. In addition, the optimal FITT factors to improve patients’ QoL remained unanswered in that study [15]. A recent meta-analysis of 34 RCTs reported a significantly improved QoL and PF of breast cancer patients after exercise. However, it was claimed that these improvements were not moderated by the exercise characteristics [16]. Another meta-analysis of eight RCTs showed a non-significant improvement in the QoL from exercise intensity or any type of exercise in breast cancer patients [17]. In contrast, a recent systematic review of 36 trials concluded that all types of exercise interventions improved the QoL in patients with breast cancer. Combined exercise was specifically cited as being more efficient than aerobic or resistance exercise alone, although no meta-analysis data were provided [7].

These systematic reviews and meta-analyses attempted to delineate the influence of exercise characteristics on patients’ clinical outcomes. However, none of these studies revealed the efficacy of individual exercise variables (i.e., type, frequency, duration, and time of session) on improving the QoL, SF, or PF of breast cancer survivors. Most importantly, the conclusions are debatable with regards to the optimal exercise variables on clinical outcomes. As such, in order to prescribe exercise intervention as an alternative medicine, it is crucial to identify the effective and optimal exercise variables that may effectively improve a patient’s clinical outcomes. Therefore, in the current systematic review and meta-analysis, we seek to examine the effects of exercise intervention on the QoL, SF, and PF of breast cancer survivors. We further sought to explore the most effective exercise characteristics (type, frequency, duration, time, and total exercise time) using a meta-regression analysis, whilst the optimal dose of exercise (time) for an improved QoL was determined using a subgroup analysis. 

## 2. Results

### 2.1. Search Results and the Selection of Studies 

**Figure 1:**
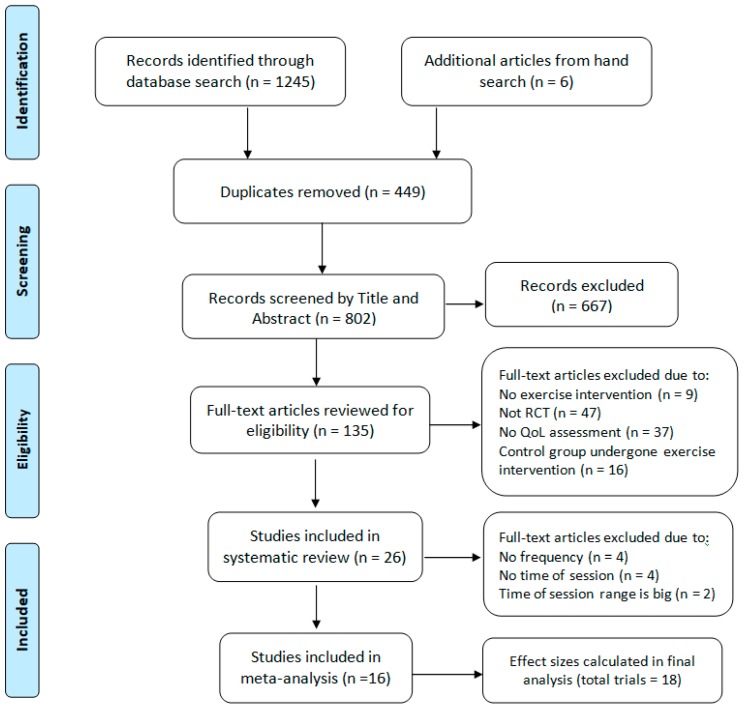
Flowchart of the study selection according to the Preferred Reporting Items for the Systematic Review and Meta-Analysis (PRISMA) method.

In the initial search, a total of 1251 articles were identified, with 1245 from electronic databases (PubMed, Web of Science, ScienceDirect, EMBASE, SportDiscus, Google Scholar) and six from a manual search (i.e., the reference list of included studies and other reviews). After removing 449 duplicates, 802 records were retrieved for further assessment. The titles and abstracts of the retrieved studies were screened using EndNote and a one by one reading, which also led to an exclusion of 667 articles. Then, the full-text of the remaining 135 articles was evaluated for the inclusion criteria, and 109 articles were excluded based on the reasons detailed in Figure 1. Finally, 26 articles that met our study’s criteria were included in the systematic review. Of these, articles with no information on exercise frequency [18,19,20,21], time of session [22,23,24,25], and with wide ranges in the time of session [26,27] were excluded. Finally, 16 studies possessing sufficient information regarding exercise characteristics and clinical outcomes were included in the meta-analysis. The detailed steps for article selection and the corresponding number of included and excluded articles in our study are presented as a flowchart in Figure 1.

### 2.2. Summary of the Included Studies 

According to the criteria, 26 articles (RCTs) were included for the systematic review, and 16 of them were used for the meta-analysis. The RCTs were intercontinental, that is, from Australia, Canada, China, England, France, Germany, Italy, Kosovo, Spain, and the United States. All the participants (1892 in total) in these RCTs were women with breast cancer. The number of patients in the trials (exercise and control) ranged from 16 to 220, and their cancer stages were from 0 to IV. For the systematic review, the included studies had conducted various types of exercise interventions, including aerobic exercise [24,25,27,28,29,30,31,32,33], resistance exercise [33,34,35,36], a combination of both [18,22,23,37,38,39,40], yoga [19,20,21,41,42], and Qigong [26,43]. The details of the patients and exercise characteristics are presented in Table 1.

For the meta-analysis, we included 16 studies that contained 18 trials, with a total of 1205 patients (exercise = 602; control = 603). Of these studies, seven trials performed aerobic exercises [28,29,30,31,32,33], four trials performed resistance exercises [33,34,35,36], four trials underwent a combination of aerobic and resistance exercises [37,38,39,40], two trials practiced yoga [41,42], and one trial performed Qigong [43], with durations of 6 to 26 weeks. The frequency of exercise varied from 2 to 7 times per week, and the length of each exercise session ranged from 25 to 90 min.

### 2.3. Patient-Reported Clinical Outcomes and Scales 

The RCTs included in our meta-analysis used well-validated questionnaires (scales) to measure the patient-reported clinical outcomes, such as the QoL, SF, and PF. For the assessment of the QoL, the European Organization for Research and Treatment of Cancer-quality of life questionnaire-C30 (EORTC-QLQ-C30) was used in five studies [33,34,35,38,39], the functional assessment of cancer therapy-general (FACT-G) scale was adopted in eight studies [28,30,32,36,37,40,42,43], the functional assessment of cancer therapy-breast (FACT-B) scale was used in two studies [29,31], and the short form-36 (SF-36) scale was employed in one study [41]. Further information on the scales used to measure the SF and PF of breast cancer survivors is presented as supplementary data (Tables S1 and S2).

### 2.4. Exercise Intervention Improves the QoL in Breast Cancer Survivors

**Figure 2:**
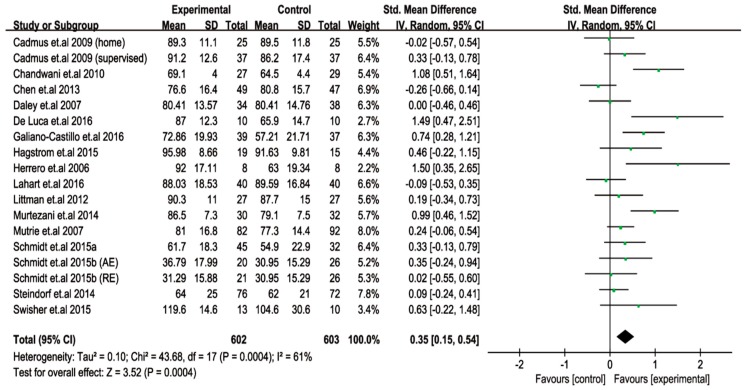
Changes in the quality of life (pooled outcomes) after exercise intervention in patients with breast cancer. SD, standard deviation; IV, inverse variation; CI, confidence internal; df, degrees of freedom.

The effect of exercise intervention on the QoL of breast cancer survivors was evaluated for 18 trials. The meta-analysis results showed that the change in QoL was extremely (*p* = 0.0004) influenced by exercise intervention, with heterogeneity: Tau^2^ = 0.10; Chi^2^ = 43.68; degrees of freedom (df) = 17; and I^2^ = 61% (Figure 2). Then, a meta-regression analysis was performed to identify the effective exercise variables that improved the QoL. Information regarding exercise type, time of session, frequency, duration, and total exercise time was entered into the meta-regression analysis. In the results, we found that except for the “time of session”, all other exercise characteristics (type, duration, frequency, and total time) were not associated with a change in QoL of breast cancer survivors. However, the “time of session” was significantly (*p* = 0.041) correlated with an improved QoL in breast cancer survivors following exercise intervention (Table 2).

### 2.5. Longer Time of Session Profoundly Improves the QoL in Women with Breast Cancer

**Figure 3:**
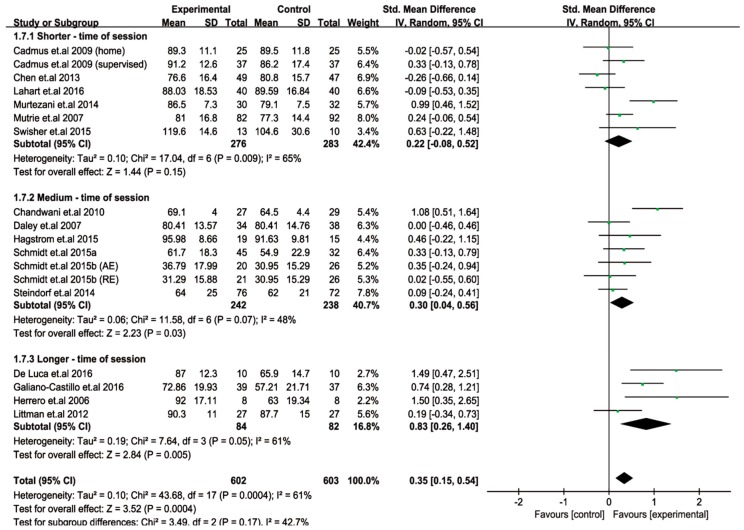
Forest plot of the quality of life (QoL) changes (subgroup analysis) with different times of exercise sessions in patients with breast cancer. SD, standard deviation; IV, inverse variation; CI, confidence internal; df, degrees of freedom.

To identify the optimal “time of session” for an improved QoL, the trials with “time of session” data were categorized into three subgroups, that is, shorter-time of session (≤45 min; 7 trials), medium-time of session (>45 to ≤60 min; 7 trials), and longer-time of session (>60 to 90 min; 4 trials). In the subgroup analysis, we found that both the sessions over the medium-time (standardized mean difference (SMD) = 0.30; I^2^ = 48%; 95% confidence internal (CI): 0.04 to 0.56, *p* = 0.03) and longer-time (SMD = 0.83; I^2^ = 61%; 95% CI: 0.26 to 1.40, *p* = 0.005) significantly improved the QoL of the breast cancer patients. However, a shorter-time of session showed no significant improvement in the QoL (SMD = 0.22; I^2^ = 65%; 95% CI: −0.08 to 0.52, *p* = 0.15). Furthermore, patients that engaged in longer exercise sessions (>60 to 90 min) appeared to achieve greater improvements (a bigger effect size) to their QoL, compared to the medium-time of session (>45 to ≤60 min) (Figure 3). 

### 2.6. Beneficial Effects of Exercise on SF amongst Breast Cancer Survivors

**Figure 4:**
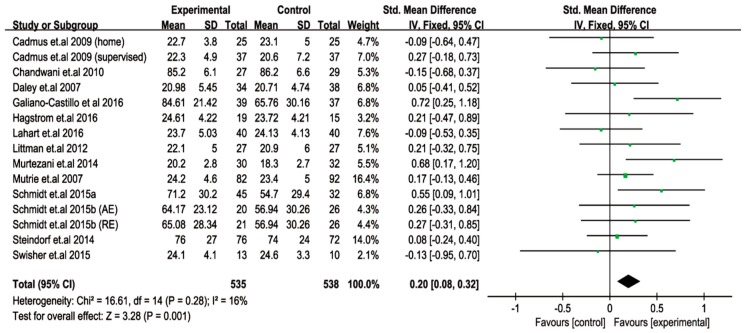
Pooled outcome of the changes in social function after exercise intervention in patients with breast cancer. SD, standard deviation; IV, inverse variation; CI, confidence internal; df, degrees of freedom.

Cancer or cancer treatment affects the social functioning of women with breast cancer. To address the influence of exercise on the SF, we included 15 eligible trials consisting of 1073 breast cancer patients (535 exercise; 538 control), and a meta-analysis was also performed. The test for the overall effect revealed that exercise interventions substantially (*p* = 0.001) improved SF in women with breast cancer. The SF outcome extremely favored exercise, citing the SMD = 0.20, I^2^ = 16%, and 95% CI: 0.08 to 0.32. Our data further indicated that none of the other specified exercise variables were correlated with improving SF in female patients (Figure 4).

### 2.7. Beneficial Effects of Exercise on PF amongst Breast Cancer Survivors

**Figure 5:**
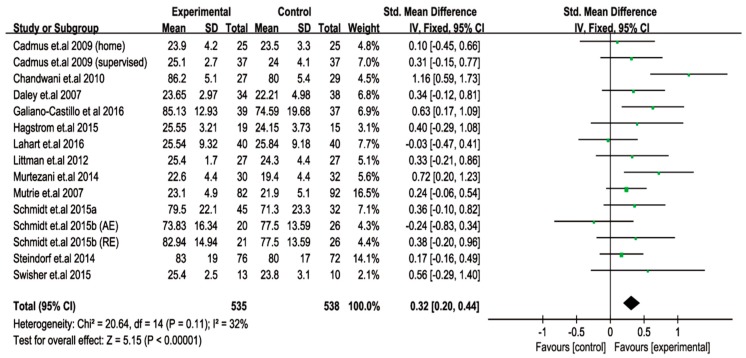
Pooled outcome of changes in physical function after exercise intervention in patients with breast cancer. SD, standard deviation; IV, inverse variation; CI, confidence internal; df, degrees of freedom.

A total of fifteen trials addressed the effects of exercise on the PF of female breast cancer survivors. The pooled outcome of meta-analysis also showed that exercise interventions improved the PF of breast cancer survivors (*p* < 0.00001). The pooled SMD of the enhanced PF was 0.32 (0.20 to 0.44), at a 95% CI, and where the I^2^ was 32% after the interventions (Figure 5).

### 2.8. Risk of Bias of Included Studies 

**Figure 6:**
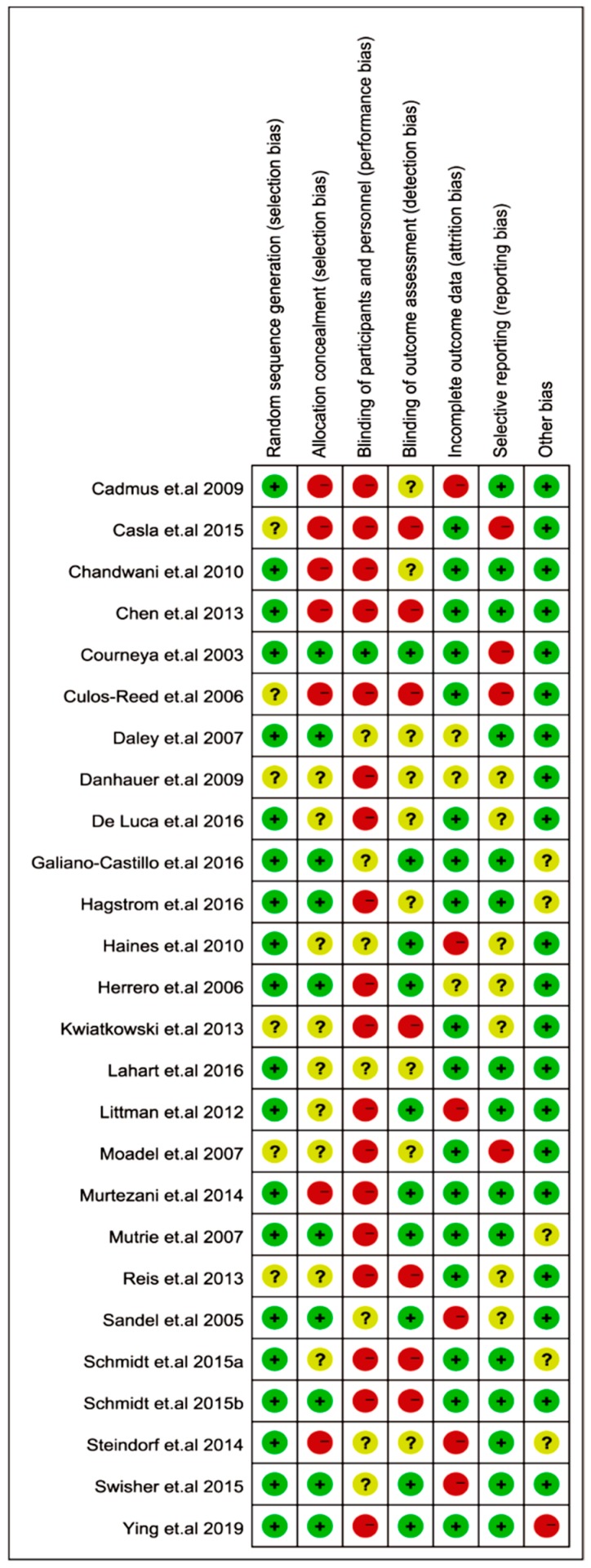
Risk of bias summary of the included studies. Green indicates a low risk of bias (+), red indicates a high risk of bias (−), and yellow indicates unclear risk (?).

The risk of biased judgments in the 26 articles is shown in Figure 6. For the selection bias, most of the studies (i.e., 20 trials) reported a low risk of random sequence generation, and seven studies were judged to have a high risk of allocation concealment. Several studies (i.e., 18 trials) were judged to have a high risk of bias in the blinding of participants towards exercise performance. Specifically, in the studies with exercise interventions, it may not feasible to blind patients in the participation of exercise. It is stated that reporting such a high risk of performance bias does not necessarily compromise the study quality [44]. Nevertheless, we identified seven trials with detection bias and six trials with an attrition bias. Only four studies were judged to have a selective reporting bias. These results indicated that most of the included studies were not found to possess a high risk of selection, adherence, attrition, and reporting bias.

## 3. Discussion

For the first time, our systematic review and meta-analysis demonstrated the influence of exercise intervention on the QoL, SF, and PF in female breast cancer survivors, and the “time of session” was identified as a crucial exercise variable for an improved QoL. Previous meta-analyses have stated that exercise promotes a better QoL in breast cancer survivors; however, the intervention effect concerning the exercise type, frequency, duration, or time of session was inconclusive [15,16,17]. In this study, we included 26 RCTs and addressed the efficacy of exercise characteristics on the patient-reported clinical outcomes. Pooled outcomes revealed that the exercise intervention significantly improved the QoL, SF, and PF of breast cancer survivors. Our subsequent meta-regression analysis showed that an improved QoL was significantly correlated with the “time of session” and not with other exercise variables (i.e., type, frequency, duration, and total exercise time). Specifically, exercise sessions for a longer-time (>60 to 90 min) appeared to be superior to the sessions for a medium-time (>45 to ≤60 min) in improving the QoL, whilst shorter-time sessions (≤45 min) had no significant effect on the QoL.

Despite the incidence of breast cancer, the number of survivors has increased [4] owing to advanced medical care and facilities. However, this greater number of survivors are still suffering from treatment-induced adverse effects, such as impairments to social functioning, physical functioning, and QoL [5,6]. In post-treatment survivors, poor subjective well-being and impaired health-related QoL, along with extreme financial distress, could be vital risk factors of mortality [45]. A study on the Brazilian female population revealed that physical inactivity was responsible for more deaths (~12%) due to breast cancer, whilst other modifiable risk factors contributed for ~5% of deaths [46]. Evidence from a cohort study emphasized the importance of exercise in the reduction of all-cancer mortality, and it was reported to extend lifespan by ~3 years in both women and men [47]. In general, exercise interventions are aimed at improving the health-related physical fitness and overall well-being of cancer survivors.

Exercise prescription has gained significant attention in recent decades as a way to overcome treatment-induced adverse effects and to promote overall well-being. Several trials have adopted various exercise protocols, and these trials have demonstrated the practical applications of such interventions in improving the clinical outcomes in breast cancer survivors [31,37,48]. Prior to establishing this evidence, patients receiving cancer treatment were advised to stay rested and to avoid physically challenging activities that may cause additional burden and fatigue, instead of alleviating the cancer-related fatigue [49]. This conceptual notion was eventually ruled-out as studies revealed the beneficial effects of exercise interventions on the improved physiological and psychological domains of cancer survivors. In our systematic review, we synthesized the data from 26 RCTs that adopted various types of exercise patterns and with different frequencies, durations, or time of session. We found that exercise intervention significantly improved the clinical outcomes, including the QoL, PF, and SF in breast cancer survivors. Although the overall response of the outcomes towards the exercise interventions was favorable, the adopted exercise protocols in the RCTs were dissimilar in their type [26,33,38,42], duration [32,43], time and dose [30,39] or frequency [28,33]. Therefore, identification of the responsible exercise moderators and the optimal time and dose of exercise is required in order to shed light on the best exercise prescription for breast cancer survivors.

QoL is a subjective and multidimensional outcome, encompassing physical function, psychological state and emotional well-being, and social well-being [50]. Much has been done to address the influence of exercise interventions on a patients’ QoL, and several studies have also found either an unchanged or slightly improved QoL in breast cancer survivors. This inconsistent result in the QoL outcome from exercise interventions might be linked to several factors associated with the exercise characteristics, such as the type, frequency, duration, or time of session [10,26,28,51]. In our meta-analysis, the pooled outcome of 18 trials showed a profoundly improved QoL following exercise, where the heterogeneity was high. Similarly, a previous meta-analysis of 34 RCTs reported an improved QoL with exercise, although this improvement was not moderated by the exercise type or any other exercise characteristics [16]. Another meta-analysis of eight RCTs found a non-significant improvement in the QoL, linked to the intensity or type of exercise [17]. On the other hand, the practice of conventional exercises, such as yoga and qigong, was also reported to improve the QoL and well-being of female breast cancer survivors [26,41,48]. It indicates that either the type or duration of exercise could influence the QoL scores in breast cancer survivors, although the optimal duration for this promising amelioration is still inconclusive.

The strength of our study was that we identified the “time of session” as a promising variable, which we considered to be responsible for the improved QoL in female breast cancer survivors. Our meta-regression analysis revealed a significant correlation between the “time of session” and an improved QoL, whereas other variables (i.e., type, frequency, duration, and total exercise time) were not associated with the QoL. A further subgroup analysis demonstrated that a shorter-time of session (≤45 min) did not contribute to better a QoL. However, both the medium-time (>45 to ≤60 min) and longer-time of sessions (>60 to 90 min) profoundly improved the QoL, where longer-time sessions showed a bigger effect size. In contrast to our findings, a prior meta-analysis found that exercise intervention durations of ≤45 min (five trials) and >45 min (three trials), had improved the QoL in patients with mixed types of cancer [15]. Another study showed that a longer time (higher dose) of either aerobic or combined exercise (50 to 60 min) was not superior to a standard aerobic exercise (25 to 30 min) program, in terms of the physical functioning of breast cancer survivors [14]. Another meta-analysis reported that neither the exercise time nor its duration significantly influenced the QoL of patients with cancer [16]. Similarly, a recent meta-analysis showed an improved QoL in cancer patients after supervised exercise, although no statistical difference was noted for the other exercise variables, including time of session [8]. Nevertheless, our findings were in agreement with the recommendations of the European Society for Clinical Nutrition and Metabolism (ESPEN), which cite that cancer patients should be engaged in exercise sessions for 45 to 60-min per day [52]. Therefore, to achieve the maximum benefits from exercise interventions, breast cancer patients should participate in any type of exercises for >45 min per session.

To the best of our knowledge, this is the first systematic review and meta-analysis to address the effects of exercise on the SF of female breast cancer survivors. Despite the etiology of cancer and cancer treatment, there is still a limited understanding of cancer-related consequences, especially of patients’ social domains. Either the diagnosis or treatment can cause sequential damages to the social functioning of women with breast cancer [53]. In the overall health-related QoL, family well-being and social well-being are the most considerable social domains for female breast cancer patients. Therefore, it is necessary to study the impacts of cancer on the family and social relationships following diagnosis, treatment, and during survival [54]. Group exercise interventions were said to foster an improved overall QoL in survivors, because the group exercises provided access to other survivors, and therefore could address the issues related to stained social relationships, stigma, and isolation [55]. In view of this, a meta-analysis using mixed types of cancer reported improved SF in cancer patients after exercise interventions. To be specific, the improved SF was observed with more exercise frequency (3–5 times/week) and with shorter workouts, but this effect was not observed with longer workouts [15]. Another meta-analysis of nine trials attempted to address the association between exercise and social well-being in breast cancer survivors. However, we noticed a discrepancy between that study’s explanations and the pooled data. In addition, the role of exercise variables was not fully disclosed and the data were not extracted from several eligible studies [51]. In our meta-analysis, we included all eligible studies (15 trials) and we found an extremely improved SF after exercise. However, this beneficial effect might not be correlated with the exercise characteristics, based on the heterogeneity.

Next, we found that exercise interventions substantially improved the PF of female cancer survivors. Previous meta-analyses have also indicated that physical activity was correlated with an improved PF and decreased fatigue symptoms in breast cancer survivors [17,56]. A recent meta-analysis by Juvet and colleagues [57] concluded that there were improvements to the PF in breast cancer trials with exercise intervention. The study’s subgroup analysis for aerobic, resistance, and mixed interventions showed no significant group differences. However, the greater beneficial effects of mixed interventions appeared to be inconsistent with the data provided in their study [57]. In another meta-analysis, the improved physical fitness of cancer patients was said to be connected with their exercise frequency (i.e., 5 times/week) and the duration of each intervention (shorter and longer) [15]. However, with a relatively higher number of studies (15 trials), we found no involvement of exercise characteristics on the improved PF. Furthermore, the pooled outcome from our study was consistent with previous meta-analyses and RCTs, which affirmed the benefits of exercise on an improved PF in cancer survivors. It is interesting to note that improved PF in breast cancer survivors was not superior with a higher dose of aerobic or combined exercise as compared to a standard dose of aerobic exercise [14]. Moreover, other meta-analyses have revealed that an improved PF was not moderated by the patient’s demographic, clinical, and exercise characteristics or the FITT factors [8,16]. Considering these points, in order to maximize the benefits of exercise interventions on PF, the type of exercise with the most optimal frequency, duration, or time still needs to be further established.

### Significance of Exercise Intervention, Scales, and the Included Trials 

To address the importance of the “time of session” in cancer survivors, one study showed an improved QoL with both a shorter and longer “time of sessions” [15], whilst another study claimed no influence of “time of session” on the QoL [16]. Our study found that the “time of session” is primarily associated with an improved QoL in breast cancer survivors. The improved PF, SF, and overall QoL in cancer survivors following exercise intervention could be explained by several possible aspects. Exercise has been shown to increase the lean body mass of breast cancer patients, and decrease the fat percentage, body mass index, and insulin level with improved muscle strength and cardiopulmonary functions [25,51]. Moreover, by participating in group or supervised exercise programs, patients can interact with the researcher or other patients that are facing similar issues. This setting allows patients to obtain health advice on their overall well-being, and it also helps to decrease their sense of isolation, social stigma, as well as improve their self-esteem [55,58]. The clinical outcomes reported in our study were self-reported and measured using various scales, including the EORTC-QLQ-C30, FACT-G, FACT-B, and SF-36. These self-reported questionnaires and scales are designed to provide robust and meaningful measurements, which are determined by objectivity, reliability, and validity. All the scales used in the included trials were well-validated, feasible, and are widely accepted in cancer clinical trials [59,60,61,62]. The empirical evidence from previous studies and the data from our meta-analysis emphasized that the implementation of exercise interventions is crucial in ameliorating the clinical outcomes in breast cancer survivors. Based on the location of the included studies, it might not be irrelevant to state that most of these RCTs were from Europe and North America. Therefore, we assume that this observation not only indicates the higher breast cancer survival rates in those regions, but it also implies the need for significant efforts and studies targeted towards the Asian region, where post-treatment cancer rehabilitation is relatively lower than the incidence.

## 4. Materials and Methods 

### 4.1. Search Strategy and the Identification of Studies

Comprehensive article searches were performed using the PubMed (Medline), Web of Science, ScienceDirect, EMBASE, SportDiscus, and Google Scholar databases to identify the relevant articles (until February 2019). The keywords used for the article search were, “breast cancer”, OR “breast tumor”, OR “breast neoplasms”, OR “breast carcinoma”, AND “exercise”, OR “aerobic exercise”, OR “aerobic training”, OR “resistance exercise”, OR “resistance training”, OR “strength training”, OR “physical activity”, OR “kinesitherapy”, OR “motor activity”, OR “sports”, OR “yoga”, OR “Tai chi”, OR “Qigong”, AND “quality of life”, OR “QoL”, OR “outcomes”. The keywords denoting breast cancer and exercise were separately used again to search for studies with “social function” and “physical function” outcome measures. In addition, a manual search was conducted using the reference list of retrieved articles, systematic reviews, and meta-analyses to identify the relevant studies. 

### 4.2. Selection Criteria

Two investigators (Feng Hong and Weibing Ye) independently screened the titles and abstracts of all the identified articles, and the duplicates were removed. The full-text articles were then evaluated for inclusion in the systematic review and the meta-analysis. The inclusion criteria were as follows: (1) All the studies were RCTs; (2) the participants were adults diagnosed with breast cancer; (3) the experimental group (breast cancer patients) had undergone any type of exercise intervention, whilst the control group (breast cancer patients) had not undergone any exercise intervention; (4) the trials measured the QoL, PF, SF, or all these factors of the cancer patients; (5) the data of the outcomes was provided before and after the interventions; and (6) the study was published in English. The exclusion criteria were: (1) Inadequate statistical information or poor quality information; (2) insufficient information about the exercise characteristics; (3) the trials included other types of cancer patients or the studies were without a control trial; and (4) the data were provided as mean change within the trial. 

Two of the authors (Feng Hong and Weibing Ye) carefully reviewed the studies, and then selected the articles that met the inclusion criteria. Then, a further in-depth review and additional information on the clinical outcomes and exercises was provided by other authors (Chia-Hua Kuo, Yong Zhang, and Yongdong Qian). Potential discrepancies regarding the inclusion or exclusion of the articles were discussed and resolved by another author (Mallikarjuna Korivi). The article review and selection process was performed using the Preferred Reporting Items for Systematic Review and Meta-Analysis (PRISMA) guidelines [63], and a detailed flowchart was provided (Figure 1).

### 4.3. Quality Assessment

The quality assessment procedure for the included RCTs was performed using the Cochran Collaboration risk of bias tool as in a previous study [64]. Each included study was evaluated for the source of bias, including: (1) random sequence generation and allocation concealment (selection bias); (2) blinding of study participants and personnel (performance bias); (3) blinding of outcome assessments (detection bias); (4) incomplete outcome data (attrition bias); (5) selective reporting (reporting bias); and (6) other sources of bias. The quality of each domain was rated as “low risk”, “high risk”, or “unclear”, and they were indicated using green (+), red (−), and yellow (?), respectively. The quality assessment of the trials was independently performed by two of the three researchers, and then compared (Feng Hong, Weibing Ye, and Yongdong Qian). The disagreements were resolved by discussing with another review author (Mallikarjuna Korivi). 

### 4.4. Data Extraction

Data from all the included trials were extracted by two independent authors (Feng Hong and Weibing Ye), and they were verified by another review author (Mallikarjuna Korivi). All the data were presented as mean with standard deviations (SD). Any data provided as standard error in the trials were converted to SD. Details of the included articles, such as authors, year, and country of publication, were recorded. Demographics of the patients (i.e., mean age, sample size, and cancer stage), as well as the characteristics of the exercise (i.e., type, time of session, frequency, duration, and total exercise time) were extracted from the included trials. The type of questionnaire used to determine the outcome measures (QoL, PF, SF) was also obtained, and the details are presented in Table S1.

### 4.5. Outcome Measures

All the clinical outcomes, including the QoL (general health, global, and overall QoL), PF, and SF, were self-reported. The general health, PF, and SF subscales from the generic short-form 36 (SF-36) [61] were used as the measures for self-reported QoL, self-reported PF, and self-reported SF, respectively. The global QoL, PF, and SF scales from the disease-specific European Organization for Research and Treatment of Cancer (EORTC) QLQ-C30 questionnaire [65] and the cancer rehabilitation evaluation system short form [66] were used as the measures for self-reported QoL, self-reported PF, and self-reported SF. The total scores from the functional assessment of cancer therapy (FACT-G, FACT-B) [62,67] were used as a measure for the self-reported QoL, whilst the physical well-being scale was used as a measure of the self-reported PF, and the social and family well-being scale was used as a measure of SF.

### 4.6. Subgroup Division and Analysis

The identified trials with sufficient “time of session” data were categorized into three subgroups, including the shorter-time of session (≤45 min), medium-time of session (>45 to ≤60 min), and longer-time of session (>60 to 90 min). This division was based on the time of each exercise session that the cancer patients performed during the course of intervention. The European Society for Clinical Nutrition and Metabolism (ESPEN) has recommended that cancer patients should engage in 45 to 60 min exercise sessions per day [59]. Based on the ESPEN recommendations and several other studies, we intended to examine the effects of shorter- and longer-times of exercise sessions on the clinical outcomes. Other exercise characteristics (i.e., type, frequency, duration, and total exercise time) were not correlated with the change of outcomes in patients with breast cancer.

### 4.7. Statistical Analysis

The Cochrane Collaboration’s Review Manager (RevMan 5.3., Copenhagen, Denmark) program was used to analyze the effects of exercise characteristics on the clinical outcome measures (QoL, SF, PF) in breast cancer survivors. Owing to the different measurement scales, we calculated the standardized mean difference (SMD) at 95% confidence intervals (CI). The I^2^ statistic was reported as an indicator of heterogeneity, with I^2^ ≥50% representing high heterogeneity and I^2^ <50% representing low heterogeneity. If the heterogeneity was low, the fixed-effects model was used for the meta-analysis. If heterogeneity was high, then the random-effects model was used for the meta-analysis. Based on the heterogeneity significance (pooled outcome), meta-regression analysis was performed to identify the correlations between the exercise characteristics (type, time, frequency, duration, and total exercise time) and the outcome measures (QoL, SF, PF) of breast cancer patients. We used the STATA version 12 (StataCorp, College Station, TX, USA) for the meta-regression analysis. In the analysis, the exercise “time of session” was found to be associated with an improved QoL. Hence, the eligible trials containing “time of session” data were assigned into three subgroups, as described above. Then, the subgroup analysis was conducted to identify the effective “time of exercise session” to improve the QoL in female breast cancer patients.

## 5. Conclusions

Our findings convincingly demonstrated that exercise intervention (of any type) is beneficial to improving the QoL, SF, and PF of female breast cancer survivors. However, the improved QoL from exercise intervention was specifically associated with the length of the exercise session. Participation in the exercise sessions for more than 45 min (medium- or longer-time) effectively improved the QoL, whilst a shorter-time of session (<45 min) did not contribute to a significant improvement. Our findings suggest that the “time of session” could be the most decisive factor in improving the overall QoL in cancer survivors. Therefore, prescribing exercise programs with >45 min per session would be a promising approach to promoting the overall health-related QoL of breast cancer survivors.

## Figures and Tables

**Table 1:** Characteristics of the included studies.

Study	Country	Age (Years)		Sample (n)		Cancer Stage	Exercise Type	Time of Session (min)	Frequency (t/wk)	Duration (Weeks)	Outcome
		Exercise	Control	Exercise	Control						
Ying et al. 2019 [26]	China	1n: <40 36n: 40–60 9n >60	2n:< 40 28n: 40–60 10n: >60	46	40	Ⅰ–Ⅲ	Qigong	S:60, H:20	7	26	QoL, PWB, S/FWB
De Luca et al. 2016 [37]	Italy	50.2 ± 9.7	46.0 ± 2.8	10	10	Ⅰ–Ⅲ	AE + RE	90	2	24	QoL
Galiano-Castillo et al. 2016 [38]	Spain	47.4 ± 9.6	49.2 ± 7.9	39	37	Ⅰ–Ⅲ	AE + RE	90	3	8	QoL, PF, SF
Hagstrom et al. 2016 [36]	Australia	51.2 ± 8.5	52.7 ± 9.4	19	15	Ⅰ–Ⅲ	RE	60	3	16	QoL, PWB, S/FWB
Lahart et al. 2016 [32]	England	52.4 ± 10.3	54.7 ± 8.3	40	40	Ⅰ–Ⅲ	AE	30	3–7	26	QoL, PWB, S/FWB
Casla et al. 2015 [22]	Spain	45.91 ± 8.21	51.87 ± 8.21	44	45	Ⅰ–Ⅲ	AE + RE	(nr)	2	12	QoL
Schmidt et al. 2015a [34]	Germany	52.2 ± 9.9	53.3 ± 10.2	45	32	Ⅰ–Ⅲ	RE	60	2	12	QoL, PF, SF
Schmidt et al. 2015b [33]	Germany	AE:56 ± 10.15 RE:53 ± 12.55	54 ± 11.19	AE:20 RE:21	26	(nr)	AE RE	60	2	12	QoL, PF, SF
Swisher et al. 2015 [31]	United States	43–65	36–71	13	10	Ⅰ–Ⅲ	AE	30	5	12	QoL, PWB, S/FWB
Murtezani et al. 2014 [30]	Kosovo	53 ± 11	51 ± 11	30	32	Ⅰ–Ⅲ	AE	25–45	3	10	QoL, PWB, S/FWB
Steindorf et al. 2014 [35]	Germany	55.2 ± 9.5	56.4 ± 8.7	76	72	0–Ⅲ	RE	60	2	12	QoL, PF, SF
Chen et al. 2013 [43]	China	45.3 ± 6.3	44.7 ± 9.7	49	47	0–Ⅲ	Qigong	40	5	6	QoL
Kwiatkowski et al. 2013 [23]	France	51.8 ± 8.7	52.3 ± 10.1	113	107	(nr)	AE + RE	(nr)	7	2	QoL, PF, SF
Reis et al. 2013 [27]	United States	54 ± 11.1	59 ± 10.7	12	17	(nr)	AE	20–60	3	12	QoL, PWB, S/FWB
Littman et al. 2012 [42]	United States	60.6 ± 7.1	58.2 ± 8.8	27	27	0–Ⅲ	Yoga	75	5	26	QoL, PWB, S/FWB
Chandwani et al. 2010 [41]	United States	51.39 ± 7.97	40.2 ± 9.96	27	29	0–Ⅲ	Yoga	60	3	12	QoL, PF, SF
Haines et al. 2010 [18]	Australia	55.9 ± 10.5	59.5 ± 13.3	33	32	(nr)	AE + RE	(nr)	(nr)	24	QoL, PF, SF
Cadmus et al. 2009 [28]	United States	H:54.5 ± 8.2 S:56.5 ± 9.5	H:54 ± 10.9 S:55 ± 7.7	H:25 S:37	H:25 S:37	0–Ⅲ	AE	30	5	26	QoL, PWB, S/FWB
Danhauer et al. 2009 [19]	United States	54.3 ± 9.6	57.2 ± 10.2	13	14	Ⅰ–Ⅳ	Yoga	70	(nr)	10	QoL, PWB, S/FWB
Daley et al. 2007 [29]	England	51.6 ± 8.8	51.1 ± 8.6	34	38	(nr)	AE	50	3	8	QoL, PWB, S/FWB
Moadel et al. 2007 [20]	United States	55.11 ± 10.07	54.23 ± 9.8	45	26	Ⅰ–Ⅲ	Yoga	(nr)	(nr)	12	QoL, PWB, S/FWB
Mutrie et al. 2007 [40]	England	51.3 ± 10.3	51.8 ± 8.7	82	92	(nr)	AE + RE	45	3	12	QoL, PWB, S/FWB
Culos-Reed et al. 2006 [21]	Canada	51.18 ± 10.33		18	18	(nr)	Yoga	75	(nr)	7	QoL
Herrero et al. 2006 [39]	Spain	50 ± 5	51 ± 10	8	8	Ⅰ–Ⅱ	AE + RE	90	3	8	QoL
Sandel et al. 2005 [24]	United States	59.7 ± 9.8	59.5 ± 13.3	19	19	(nr)	AE	(nr)	1–2	12	QoL
Courneya et al. 2003 [25]	Canada	59 ± 5	58 ± 6	24	28	Ⅰ–Ⅲ	AE	(nr)	3	12	QoL, PWB, S/FWB

Note: t/wk, times/week; H, home-based; S, supervised; AE, aerobic exercise; RE, resistance exercise; AE + RE, combination of aerobic and resistance exercise; nr, not reported; QoL, quality of life; SF, social function; PF, physical function; PWB, physical well-being; S/FWB, social/family well-being.

**Table 2:** Meta-regression analysis to identify the effective exercise moderators on an improved quality of life in breast cancer survivors.

Exercise Characteristics	Coefficient	Standard Error	T Value	*p*-Value
Type of exercise	−0.3471926	0.3498219	−0.99	0.339
Time of session	0.0121459	0.0054789	2.22	0.041 *
Frequency	−0.1384031	0.0839449	−1.65	0.119
Duration	−0.006877	0.0171193	−0.40	0.693
Total time of exercise	−0.0000111	0.0000583	−0.19	0.851

* Represents a significant correlation between the quality of life and exercise variable (time of session).

## Supplementary Materials

The following are available online at https://www.mdpi.com/2072-6694/11/5/706/s1, Table S1: Characteristics of the 18 trials, Table S2: Assessment scales and studies.
