# Supplementary material for: Exercise Intervention Improves Clinical Outcomes, but the “Time of Session” is Crucial for Better Quality of Life in Breast Cancer Survivors: A Systematic Review and Meta-Analysis
Source: Cancers (Basel). 2019 May 22;11(5):706. doi: 10.3390/cancers11050706 (PMC6562879; doi:10.3390/cancers11050706)
Supplement: Supplementary file 1 [file cancers-11-00706-s001.pdf]

Supplementary materials

Table S1. Characteristics of 18 trials.

Study	Country	Age (Years)		Sample		Cancer Stage	Exercise Type	Time of Session (min)	Frequency (t/wk)	Duration (Weeks)	Outcome	Questionnaire
		Exercise	Control	Exercise	Control							
De Luca et.al 2016 [37]	Italy	50.2 ± 9.7	46.0 ± 2.8	10	10	I–III	AE + RE	90	2	24	QoL	FACT-G
Galiano-Castillo et.al 2016 [38]	Spain	47.4 ± 9.6	49.2 ± 7.9	39	37	I–III	AE + RE	90	3	8	QoL, PF, SF	QLQ-C30
Hagstrom et.al 2016 [36]	Australia	51.2 ± 8.5	52.7 ± 9.4	19	15	I–III	RE	60	3	16	QoL, PWB, S/FWB	FACT-G
Lahart et.al 2016 [32]	England	52.4 ± 10.3	54.7 ± 8.3	40	40	I–III	AE	30	3-7	26	QoL	FACT-G
Schmidt et.al 2015a [34]	Germany	52.2 ± 9.9	53.3 ± 10.2	45	32	I–III	RE	60	2	12	QoL, PF, SF	QLQ-C30
Schmidt et.al 2015b (AE) [33]	Germany	56 ± 10.15	54 ± 11.19	20	26	(nr)	AE	60	2	12	QoL, PF, SF	QLQ-C30
Schmidt et.al 2015b (RE) [33]	Germany	53 ± 12.55	54 ± 11.19	21	26	(nr)	RE	60	2	12	QoL, PF, SF	QLQ-C30
Swisher et.al 2015 [31]	USA	43-65	36-71	13	10	I–III	AE	30	5	12	QoL, PWB, S/FWB	FACT-B
Murtezani et.al 2014 [30]	Kosovo	53 ± 11	51 ± 11	30	32	I–III,	AE	25–45	3	10	QoL, PWB, S/FWB	FACT-G
Steindorf et.al 2014 [35]	Germany	55.2 ± 9.5	56.4 ± 8.7	76	72	0–III	RE	60	2	12	QoL, PF, SF	QLQ-C30
Chen et.al 2013 [43]	China	45.3 ± 6.3	44.7 ± 9.7	49	47	0–III	Qigong	40	5	6	QoL	FACT-G
Littman et.al 2012 [42]	USA	60.6 ± 7.1	58.2 ± 8.8	27	27	0–III	Yoga	75	5	26	QoL, PWB, S/FWB	FACT-G
Chandwani et.al 2010 [41]	USA	51.39 ± 7.97	40.2 ± 9.96	27	29	0–III	Yoga	60	3	12	QoL, PF, SF	SF-36
Cadmus et.al 2009 (home) [28]	USA	54.5 ± 8.2	54.0 ± 10.9	25	25	0–III	AE	30	5	26	QoL, PWB, S/FWB	FACT-G
Cadmus et.al 2009 (supervised) [28]	USA	56.5 ± 9.5	55.1 ± 7.7	37	37	0–III	AE	30	5	26	QoL, PWB, S/FWB	FACT-G
Daley et.al 2007 [29]	England	51.6 ± 8.8	51.1 ± 8.6	34	38	(nr)	AE	50	3	8	QoL, PWB, S/FWB	FACT-B

Mutrie et.al 2007 [40]	England	51.3 ± 10.3	51.8 ± 8.7	82	92	(nr)	AE + RE	45	3	12	QoL, PWB, S/FWB	FACT-G
Herrero et.al 2006 [39]	Spain	50 ± 5	51 ± 10	8	8	I–II	AE + RE	90	3	8	QoL	QLQ-C30

t/wk, times/week; AE, aerobic exercise; RE, resistance exercise; AE + RE, combination of aerobic and resistance exercise; NR, not reported; QoL, quality of life; SF, social function; PF, physical function; PWB, physical well-being; S/FWB, social/family well-being; FACT-B, functional assessment of cancer therapy-breast; FACT-G, functional assessment of cancer therapy-general; SF-36, health survey short form-36; QLQ-C30, European Organization for Research and Treatment of Cancer Quality of Life Questionnaire.

Table S2. Assessment scales/questionnaires used in the included studies.

Assessment Scales	Studies	
EORTC-QLQ-C30	Schmidt et.al 2015b [33]; Steindorf et.al 2014 [35];	Schmidt et.al 2015a [34];
	Herrero et.al 2006 [39];	Galiano-Castillo et.al 2016 [38]
FACT-G	Cadmus et.al 2009 [28]; Hagstrom et.al 2016 [36]; Mutrie et.al 2007 [40]; Chen et.al 2013 [43];	Murtezani et.al 2014 [30]; De Luca et.al 2016 [37]; Littman et.al 2012 [42]; Lahart et.al 2016 [32]
FACT-B	Swisher et.al 2015 [31];	Daley et.al 2007 [29]
SF-36	Chandwani et.al 2010 [41]	

EORTC-QLQ-C30, European Organization for Research and Treatment of Cancer Quality of Life Questionnaire; FACT-G, functional assessment of cancer therapy-general; FACT-B, functional assessment of cancer therapy-breast; SF-36, health survey short form-36.
